# Seizure-related differences in biosignal 24-h modulation patterns

**DOI:** 10.1038/s41598-022-18271-z

**Published:** 2022-09-05

**Authors:** Solveig Vieluf, Rima El Atrache, Sarah Cantley, Michele Jackson, Justice Clark, Theodore Sheehan, William J. Bosl, Bo Zhang, Tobias Loddenkemper

**Affiliations:** 1 Division of Epilepsy and Clinical Neurophysiology, Boston Children’s Hospital, Harvard Medical School, 300 Longwood Ave, Boston, MA 02115 USA; 2 Institute of Sports Medicine, Paderborn University, Warburger Str. 100, 33098 Paderborn, Germany; 3 Department of Neurology, Boston Children’s Hospital, Harvard Medical School, 300 Longwood Ave, Boston, MA 02115 USA; 4 Computational Health Informatics Program, Boston Children’s Hospital, Harvard Medical School, 401 Park Drive, Landmark Building, Boston, MA 02115 USA; 5 Health Informatics Program, University of San Francisco, San Francisco, CA 94117 USA

**Keywords:** Biomarkers, Neurology, Epilepsy

## Abstract

A seizure likelihood biomarker could improve seizure monitoring and facilitate adjustment of treatments based on seizure risk. Here, we tested differences in patient-specific 24-h-modulation patterns of electrodermal activity (EDA), peripheral body temperature (TEMP), and heart rate (HR) between patients with and without seizures. We enrolled patients who underwent continuous video-EEG monitoring at Boston Children’s Hospital to wear a biosensor. We divided patients into two groups: those with no seizures and those with at least one seizure during the recording period. We assessed the 24-h modulation level and amplitude of EDA, TEMP, and HR. We performed machine learning including physiological and clinical variables. Subsequently, we determined classifier performance by cross-validated machine learning. Patients with seizures (n = 49) had lower EDA levels (p = 0.031), EDA amplitudes (p = 0.045), and trended toward lower HR levels (p = 0.060) compared to patients without seizures (n = 68). Averaged cross-validated classification accuracy was 69% (AUC-ROC: 0.75). Our results show the potential to monitor and forecast risk for epileptic seizures based on changes in 24-h patterns in wearable recordings in combination with clinical variables. Such biomarkers might be applicable to inform care, such as treatment or seizure injury risk during specific periods, scheduling diagnostic tests, such as admission to the epilepsy monitoring unit, and potentially other neurological and chronic conditions.

## Introduction

Currently, the standard treatment outcome measure in epilepsy is seizure reduction or prevention. Therefore, effective epilepsy treatment relies on determining seizure frequency, and ideally preemptive seizure likelihood assessment. Seizure diaries are widely used for seizure tracking and show potential for seizure forecasting based on cyclic patterns^1–3^. Furthermore, seizure likelihood assessments have been accomplished in adults with neuro-surgically implanted intracranial electrodes^4^, indicating that detectable physiological changes precede seizures. However, a less invasive seizure forecasting method is needed.

Recent developments in wearable technologies now enable improved seizure tracking based on autonomic manifestations of seizures. Autonomic nervous system (ANS) changes occur frequently in children with epilepsy and may serve as a potential biomarker for seizure risk^5–7^. Specifically, electrodermal activity (EDA), an autonomic marker for sympathetic skin activity, exhibits unique properties in the setting of seizures and thus may be used to determine seizure likelihood when combined with other ANS modalities^8–10^ and potentially additional clinical information. In our previous study, group-specific analysis of continuously monitored EDA showed a 24-h pattern of change^11^. The modulated pattern differed between patients with and without seizures. On a group level, recordings with seizures had lower EDA levels and amplitudes than recordings without seizures. Our previous study^11^ derived 24-h patterns from patient groups and thus was inherently limited to a group difference analysis. In this study, we present an individual-based analysis of 24-h patterns. To depict the ANS multidimensionally, we also include heart rate (HR) in our analyses in addition to EDA and peripheral body temperature (TEMP). An individualized modeling approach allows for testing biomarker performance as an essential next step to evaluate the clinical relevance.

This study aims to validate seizure-related individual differences in 24-h modulation patterns in autonomic recordings, including EDA, TEMP, and HR. Additionally, we aimed to differentiate between patient groups with and without seizures by combining physiological and clinical variables. We enrolled patients undergoing continuous video-EEG monitoring and asked patients to wear an E4 biosensor, utilizing video-EEG recorded seizures as the gold standard for seizure detection. Based on our previous group-based 24-h modeling, we expected lower modulation levels and amplitudes for patients with seizures.

## Results

We included 117 patients with epilepsy diagnoses and complete clinical data. Forty-nine patients formed the seizure group (8 patients with FIAS, 26 patients with GTCS, and 15 patients with both FIAS and GTCS), and 68 patients formed the no-seizure group (see Supplement 1 for inclusion diagram). Demographic and clinical characteristics are summarized in Table 1 for each subgroup. Additional seizure information is presented in Supplement 2 for the seizure group.

**Table 1:** Group-wise demographic and clinical characteristics of all patients included in 24-h EDA pattern analysis.

	No-seizure group (n = 68)	Seizure group (n = 49)	p-value
**Sex**			0.098
Male	30 (44.1%)	30 (61.2%)	
Female	38 (55.9%)	19 (38.8%)	
**Age at enrollment**			0.019
In years, median (IQR, p25-p75)	9.4 (8.4, 7.0–15.4)	13.2 (7.2, 9.6–16.8)	
Age at First Seizure			0.021
In years, median (IQR, p25-p75)	3.5 (5.9, 1.1–7.0)	7.0 (8.5, 2.0–10.5)	
**Seizure frequency (estimated per 30 days)**			0.839
No per month (IQR, p25-p75)	4.0 (20.3, 0.7–20.9)	4.0 (9.7, 1.0–10.7)	
**Etiology of epilepsy**			0.219
Structural	22 (32%)	23 (47%)	
Unknown	36 (53%)	20 (41%)	
Genetic	6 (9%)	3 (6%)	
Immune	1 (2%)	2 (4%)	
Infectious	0 (0%)	1 (2%)	
Metabolic	0 (0%)	0 (0%)	
Not Reported	3 (4%)	0 (0%)	
Interictal EEG*			
Normal	18 (26%)	4 (8%)	0.097
Spikes	55 (81%)	43 (88%)	0.320
Focal Slowing	14 (21%)	23 (47%)	0.002
Generalized Slowing	10 (15%)	0 (0%)	0.005
**MRI Findings**			0.050
Normal	20 (29%)	6 (12%)	
Abnormal	33 (49%)	25 (51%)	
Not done/not available	15 (22%)	18 (37%)	
**Reduction of at least 1 ASM during EMU stay**			0.001
Yes	18 (26%)	31 (63%)	
No	46 (68%)	18 (37%)	
Not available	4 (6%)	0 (0%)	
Wristband Location**			0.350
Left wrist	18	13	
Left ankle	18	18	
Left unavailable	3	0	
Right wrist	17	13	
Right ankle	11	4	
Right unavailable	1	0	
Unavailable	0	1	
**Recording length in hours (after 10 min segment exclusion)**			0.014
Mean (p25-p75)	20.94 (18.5–23.0)	19.33 (17.0–21.3)	

We calculated Chi-square tests for categorical and Mann–Whitney-U tests for continuous variables to compare groups.

*Patients with interictal abnormalities may have had more than one abnormality.

**If two wristbands were placed, we included one recording and preferred left wrist, over right wrist, over ankle recordings.

**Figure 1:**
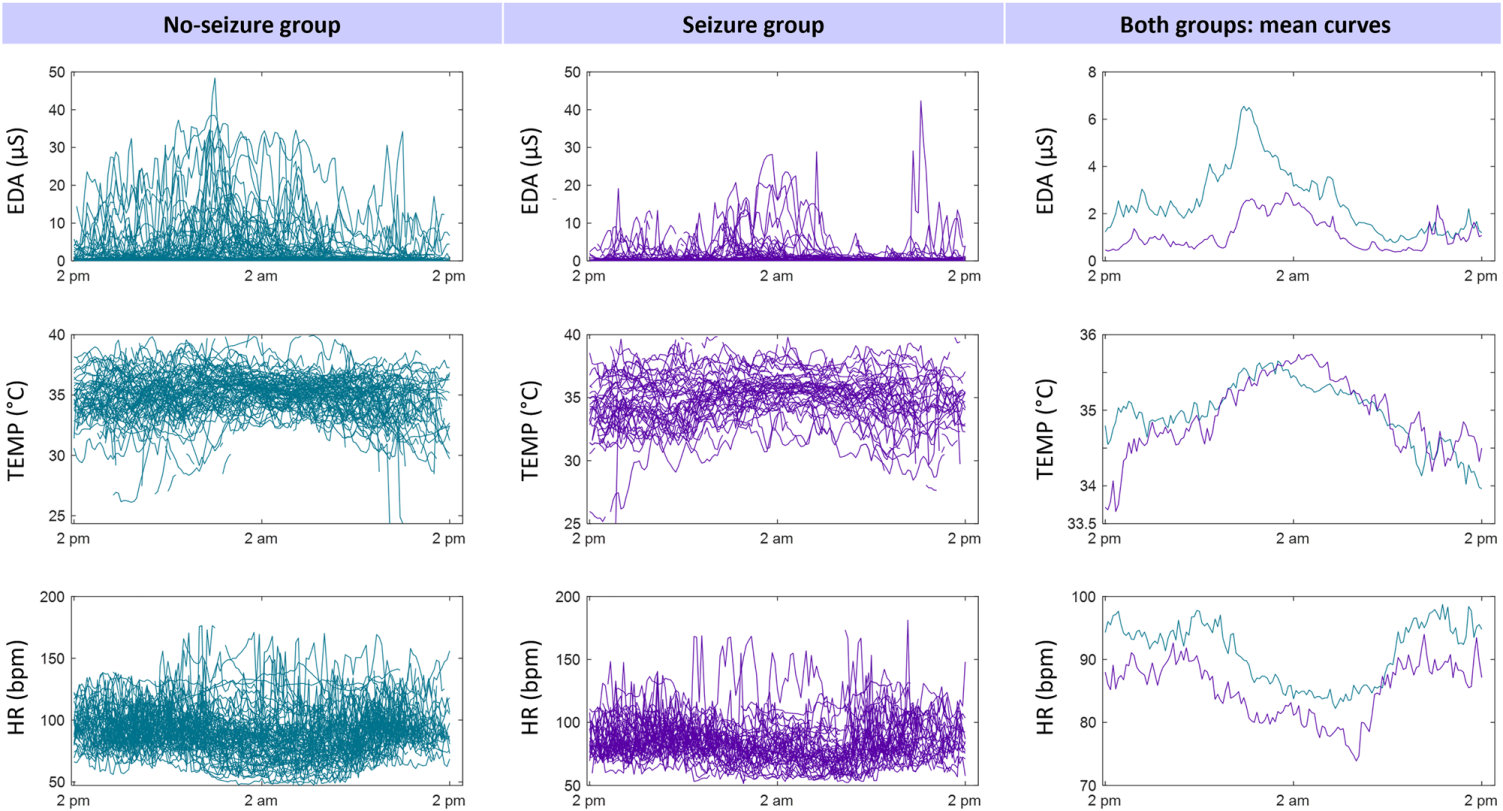
Individual recordings of EDA, TEMP, HR (from top to bottom) averaged over 10-min segments of no-seizure (teal left panel) and seizure patients (purple middle panel) are displayed over 24 h. The right panel shows the mean curves of respective autonomic modalities for no-seizure (green) and seizure (purple) patient groups.

Individual recordings revealed a pattern of change over time for both groups and all modalities (Fig. 1). While EDA and TEMP peaked at night, HR decreased during the night and showed a trough in the morning hours, starting around 6 am. TEMP was peripherally recorded at the wrist or ankle and therefore varies from core temperature curves. EDA level and amplitude, as well as tendencies for HR level, showed group differences with lower values for the seizure group. Descriptive group statistics and univariate logistic regression p-values for modulation level and amplitudes of EDA, TEMP, and HR are summarized in Table 2.

**Table 2:** Group-wise summary of modulation level and amplitude of the 24-h modulation of EDA, TEMP, and HR.

	No-seizure group					Seizure group					Statistics
	Mean	SD	Median	Percentile 25	Percentile 75	Mean	SD	Median	Percentile 25	Percentile 75	P
**Level of 24-h modulation**											
EDA (µS)	2.31	3.38	1.21	0.30	2.58	1.11	1.17	0.74	0.34	1.29	**0.031**
TEMP (°C)	35..02	1.29	35.02	34.02	35.90	34.96	1.35	34.73	34.34	35.84	0.804
HR (bpm)	91.05	14.16	91.11	79.70	99.89	86.17	12.52	83.92	77.81	93.97	0.060
**Amplitude of 24-h modulation**											
EDA (µS)	5.33	6.20	3.26	0.45	8.14	3.18	4.21	1.41	0.59	4.14	**0.045**
TEMP (°C)	1.67	1.17	1.46	0.82	2.29	2.05	1.62	1.54	0.99	2.66	0.148
HR (bpm)	24.28	10.52	22.31	16.39	31.25	24.04	10.91	22.72	15.03	31.52	0.906

Mean, standard deviation (SD), median, and 25th and 75th percentiles are presented for the no-seizure and seizure groups. P = values of univariate logistic regressions are presented in statistics.

Significant values are in bold.

The differentiation of recordings with and without seizures was better than chance. On average, cross-validated machine learning models differentiate between groups with an accuracy of 0.69, a sensitivity of 0.68, a specificity of 0.69, and an AUC-ROC of 0.75 (see Supplement 3 for individual classifier performance). Label shuffling revealed that classification results significantly differ from chance (mean accuracy of 200 shuffles = 0.52; p = 0.05, for all classifiers). Feature selection embedded in the cross validation revealed an optimal parameter number of 15, meaning that all wearable and clinical data contribute to the best classification model. Wearable data alone did not classify patients. Clinical data contained relevant information and allowed classifying patients with and without seizures (see Supplement 3 for classifier performance).

*Results of a within patient comparison of EDA, TEMP, and HR levels and amplitudes are illustrated in* Supplement 4. In our data set, 14 patients had a pre-seizure and a seizure record. The 14 patients are part of the seizure group, and the seizure-free recordings were only included in the within-patient comparison and not for the group comparison. HR levels were lower in pre-seizure compared to seizure recordings (F(1,13) = 6.68, p = 0.02, η_p_^2^ = 0.34). EDA amplitudes (F(1,13) = 3.46, p = 0.09, η_p_^2^ = 0.21) trended lower in pre-seizure compared to seizure recordings. Modulation TEMP levels and amplitudes (level: F(1,13) = 0.94, p = 0.35, η_p_^2^ = 0.07; amplitude: F(1,13) = 0.81, p = 0.38, η_p_^2^ = 0.06), HR amplitude (F(1,13) = 2.01, p = 0.18, η_p_^2^ = 0.13) and EDA level (F(1,13) = 1.27, p = 0.28, η_p_^2^ = 0.09) did not differ between recordings.

## Discussion

In a previous study, we showed seizure-related differences in 24-h EDA patterns, modeled per patient group, between patients with and without seizures. The current study validated our previous finding and built on the results through patient-specific 24-h pattern modeling and the inclusion of clinical variables and HR recordings, enabling us to differentiate between patients who had one or more seizures and patients without seizures during the recording. We used patient-level analysis to test the characteristics of the 24-h pattern as a biomarker for seizure monitoring. Following a multimodal approach, we analyzed characteristics of 24-h patterns of HR, EDA, and TEMP to classify patients into those with and without seizures. Patients with seizures had lower EDA levels and amplitude and lower HR as compared to patients without seizures. Feature selection revealed that combining EDA mean levels with clinical variables produced the best model and this model differentiates between patients with and without seizures better than chance. Comparing pre-seizure to seizure recordings within the same patient suggests that changes happen before the seizure day and, consequently, physiological markers might be predictive.

24-h patterns of peripherally recorded autonomic activity differ between recordings with and without seizures within and across participants. Central regulations of circadian patterns modulate ANS activity^12^. As a result, ANS subsystem activity shows interconnected 24-h patterns that change based on the disease state^13,14^. For epilepsy patients, 24-h patterns might be affected by long- and short-term alterations of autonomic functioning. While individual seizures manifest in acute autonomic responses, recurrent seizures and changes in central structures may cause long-term changes in autonomic control and regulations^15^. Heart rate, body temperature, and EDA exhibit circadian variation. The suprachiasmatic nucleus, responsible for the body’s circadian control, guides the autonomic outputs to maintain homeostasis and the organized physiological shifts between sleep and awake. Disruption of this control may increase susceptibility to disease^16^. In healthy subjects, HR peaks early in the afternoon and drops during the night^17^. Sweating threshold and skin temperature are highest in the evening and lowest past midnight^18^. Conversely, EDA peaks past midnight and is lowest during late afternoon hours^11,19^, which is similar to the no-seizure group results. In this study, we focused on seizure-related differences in 24-h modulation patterns of autonomic activity and therefore included patients with epilepsy diagnosis only. We validated our previous finding that 24-h modulation patterns in EDA recordings show a seizure-related lower amplitude and level of the curve^11^. Moreover, the multimodal analysis revealed a lower HR level in the 24-h modulations, confirming the effects of epilepsy on cyclic regulation of the cardiorespiratory system^20,21^.

Furthermore, for a small subset of patients, the patient-specific analysis showed that HR levels are lower while EDA amplitudes tended to be higher in pre-seizure compared to seizure recordings. This result suggests HR is altered before a seizure and is a step towards understanding the 24-h modulation curve flattening as a pre- or post-ictal phenomenon and that seizure-related autonomic changes might occur on different time scales for different modalities, i.e., following a multimodal pattern.

The combination of physiological and clinical variables allows to distinguish between recordings with and without seizures. Combining 24-h pattern levels and amplitudes with select clinical variables classifies best between patient groups. Patient group classification has been mostly limited to psychogenic nonepileptic seizures and epileptic seizures across patients^23–25^. Thus, we expected peri-ictal changes to induce a pattern of change that is constant across patients. However, while some similarities exist across patients, ANS activity largely varies between and within individuals^26–28^.

Individual clinical characteristics affect ANS modulation in patients with epilepsy. By including clinical variables, we accounted for some of this variability across patients. The final classification model included sex, epilepsy diagnosis, age at first seizure, MRI findings, reduction of ASM during the hospital stay, normal EEG, spikes, and generalized slowing. Females generally have higher parasympathetic activity, whereas males have higher sympathetic surges^29^. Additionally, sex might interact with other clinical variables as well as with the physiological variables, but our data set is too small to further explore interactions or potential influence of puberty onset. The age at first seizure relates to many developmental processes and indicates the duration of epilepsy as well, which might result in the manifestation of seizures over time. Furthermore, structural brain abnormalities seen on MRI may alter or disrupt the pathways and processes of the central autonomic network^30^. The reduction of ASM is meant to induce seizures during the stay and our results illustrate this aspect. The impact of ASMs on seizure likelihood is crucial, and while we did not have detailed pharmacokinetic data, we included patients if ASMs were reduced to adjust for possible interaction, and medication effects will require additional future study. Furthermore, interictal EEG findings contribute to the classification of patients with and without seizures. Twenty-two patients had a normal EEG and were diagnosed with epilepsy. More of those patients were in the no-SZ (18) than in the SZ (4) group, meaning that a patient with a normal EEG is likely to not have a seizure. Two types of epileptiform activity, i.e., spikes and general slowing contributed to the predictive model, while focal slowing was excluded. Note that patients can have multiple interictal EEG abnormalities. Beyond the importance of EEG activity for seizure likelihood assessments, the interplay of autonomic markers and the interictal EEG activity may also be of interest for further developing seizure detection and prediction systems. Selecting and collecting the most informative physiological and clinical data will remain an ongoing process and might improve classifier performance.

Monitoring 24-h modulation patterns might contribute to seizure detection and prediction. We have shown the ability to distinguish between recordings with and without seizures based on machine learning-based classifications. As our data analysis includes full-day recordings despite seizure time during the day, we, currently, cannot determine whether the 24-h modulations are affected by pre-ictal or post-ictal changes or both. This distinction would allow for further evaluation of 24-h patterns as biomarkers for seizure detection, prediction, and forecasting. Our group-based monitoring approach could be combined with patient-specific approaches presented for seizure prediction and detection^10,22,31–33^. Estimating seizure risk based on group classification is feasible after one day of recording and without the occurrence of a seizure. These findings show great potential for the development of patient-specific approaches to individualized seizure monitoring but require obtaining recordings with multiple seizures.

The 24-h pattern biomarkers could also be combined with existing forecasting approaches. Besides physiological data, seizure diaries and spike evaluations from EEG recordings have been successfully tested as seizure forecasting tools. In the outpatient setting, seizure diary data has shown the potential to monitor seizures^34,35^. In the inpatient setting, specifically during video-EEG monitoring, spikes are valuable to seizure forecasting models^36,37^. Both approaches involve cyclic seizure patterns and may be related to the 24-h patterns we establish here. First empirical evidence showed that seizures occurred phase-locked to circadian and multi-day cycles in HR recordings^38^. As seizure patterns occur on multiple time scales^1,2^, it might be of interest to test for similar patterns in multimodal ANS recordings. Seizure monitoring systems may even further be improved by combining these ANS recordings with seizure diaries and characteristics of physiological rhythm.

Findings need to be interpreted in the setting of data acquisition, including related selection and information bias. This study is limited by the patient cohort, quality of the E4 signals, and study setting. While our patient population is robust in the context of a clinical trial, machine learning approaches require a much larger number of patients to achieve high algorithm performance and clear results. Larger sample size would allow the inclusion of additional patient variables in the forecast, which would reduce classification uncertainty. Additionally, the wide age range of the cohort may have limited feature selection. Our best model did not include age at enrollment or age at epilepsy onset, but these variables could be predictive with a larger sample size. As this is a retrospective study, clinical data collection is limited to a chart review of existing clinical notes, which induces an information bias. Collecting and analyzing additional clinical information, such as ASM type and dose, patients specific and physiological variables, as well as seizure diary information, could improve the model.

We attempted to mitigate selection bias based on enrollment of patients to the video-EEG monitoring by offering enrollment randomly to patients, but we cannot rule out that we may have selected more severely affected patients based on inpatient enrollment, and results can therefore not be generalized without additional analysis. To test for generalizability, 24-h patterns in patients with other seizure types need to be assessed. Longitudinal measurements would also be necessary for within-patient analysis, which may lead to markers for day-to-day variability in 24-h patterns. Ideally, longitudinal measurements would occur in both inpatient and outpatient settings. However, the outpatient setting might introduce recording quality challenges.

Data quality is one of the major challenges with wearable recordings. In this study, we set thresholds to detect low signal quality. For example, periods, where the device lost contact with skin, were excluded from further analysis. A standardized data quality assessment tool would help to score signal quality and could be integrated into outcome reliability estimations. Data storage and battery life are the main limiting data quality factors in wearables but can be addressed in preprocessing to select recorded ANS modalities. Furthermore, the sensors used in this study do not include a marker for respiratory changes, core body temperature, or room temperature. However, the device has the advantage that the sub-modalities are recorded synchronized at the same body position. Future fine tuning of machine learning parameters based on larger data set may be able to refine results further. Despite limitations, this study confirms seizure-related differences in modeled 24-h patterns from shorter recordings and shows that the analysis of these patterns in the longitudinal setting could have much potential in seizure monitoring.

In summary, seizure-induced changes in autonomic activity affect 24-h modulation patterns in individuals. Differences point towards lower activity and smaller deflections on a 24-h scale. Within-patient comparison validates our previous finding that ANS changes occur before seizures with different timing for different modalities. Our results show the potential to monitor epileptic seizures based on changes in 24-h patterns from wearable recordings when combined with clinical variables. Such biomarkers might have the potential for application to other neurological diseases that affect autonomic activity.

## Materials and methods

### Standard protocol approvals, registrations, and patient consent

The study was approved by the Boston Children's Hospital Institutional Review Board (IRB-P00001945). Written informed consent was obtained from all participants and/or their guardians. This research was performed in accordance with the guidelines and regulations of the Institutional Review Board at Boston Children’s Hospital and all applicable government regulations and the Declaration of Helsinki.

### Patient selection

We included prospectively enrolled patients admitted to the Long-Term Epilepsy Monitoring Unit (EMU) for video-EEG monitoring at Boston Children’s Hospital, between February 2015 and February 2021, who wore an E4 biosensor (Empatica Inc., Milan, Italy) on either wrists or ankles. We selected patients who had at least one generalized tonic–clonic seizure (GTCS) or focal impaired awareness seizure (FIAS) during video-EEG while wearing the E4 device (SZ), or who did not have seizures during video-EEG (no-SZ) (Supplement 1). We excluded patients with status epilepticus (seizures longer than 10 min for FIAS and 5 min for GTCS) and patients with incomplete data. If multiple recordings were available per patient, we included the earliest recording favoring the right body side, to equalize the number of recordings of the left and right sides of the body, as the sensor was placed more often on the left body side in this dataset. The sensor location did not differ between groups (see Table 1). For the SZ group, the recording that included at least one seizure was selected. If multiple 24-h recordings contained seizures, we selected the one with fewer seizures to maximize inter-ictal recording length.

### Data recording and quality check

The E4 sensors captured electrodermal activity (EDA, sampling rate 4 Hz), peripheral body temperature (TEMP, sampling rate 4 Hz), and heart rate (HR, sampling rate 1 Hz). The recordings started between 9 a.m. and 4 p.m. We included data up to 5 p.m. the next day. To allow wristbands to calibrate and to exclude the wristband removal time from the recording, we excluded the first 20 min and last 10 min of each recording. The recording start time was rounded up to the nearest 10-min increment (e.g., a recording start time of 9:47 a.m. was rounded to 9:50 a.m.). We performed a data quality check per 10-min segment. The quality check failed when 10-min mean values had either an EDA level lower than 0.05 µS, an HR lower than 45 bpm or higher than 200 bpm, or a TEMP lower than 20 °C or higher than 40 °C. The peripheral recordings represent a combination of ambient and body temperatures and therefore might be comparably low if the ambient temperature is low. If the data quality check failed, we excluded these segments from the analysis. After the quality check and seizure time exclusion, we excluded any remaining patients with less than 80 clean segments, to ensure that our 24-h modeling is based on a recording length of over 13 h. For SZ patients, we excluded three 10-min pre-ictal segments and six post-ictal 10-min segments, including the segment during which the seizure occurred. We evaluated a total of 18,072 segments. From those we excluded 2589 due to low data quality and 1016 due to seizures, leading to a final data set of 14,467 10-min segments. Some patients were enrolled on multiple days during the same EMU stay. For the above-mentioned analysis, we included only one recording per patient. If there were multiple recordings, we selected one with seizure, and between seizure recordings we chose the first one during the admission for the main analysis. To obtain insights into within-patient effects, we analyzed seizure-free recordings for the SZ patients, when available. Seizure-free days were recorded one or two days before the seizure day, and we modeled 24-h patterns and calculated amplitude and level for EDA, HR, and TEMP as described above. The within-patient comparison was a separate analysis testing for within-patient changes to derive a hypothesis about using 24-h patterns in seizure forecasting models. This data set did not contain enough seizure-free days after the seizure day so we could not evaluate the potential of 24-h patterns in seizure detection models.

### Clinical data collection

We collected clinical data for patients that passed the data quality check. Using clinical notes, we collected age, sex, age of first seizure, etiology of epilepsy, MRI findings, seizure frequency, reduction in anti-seizure medications (ASM) during the hospital stay, and interictal abnormalities, i.e., normal EEG, spikes, focal slowing, generalized slowing (for details see Supplement 5). Seizure frequency values were missing for 22 patients. We replaced those with the group mean to include patients for the overall analysis. Per ILAE 2017 guidelines, a board-certified epileptologist reviewed the video-EEG recordings to determine seizure type and electrographic seizure onset and offset times^39^. We classified tonic–clonic seizures of focal and generalized onset as GTCS.

### Data analysis and statistics

**Figure 2:**
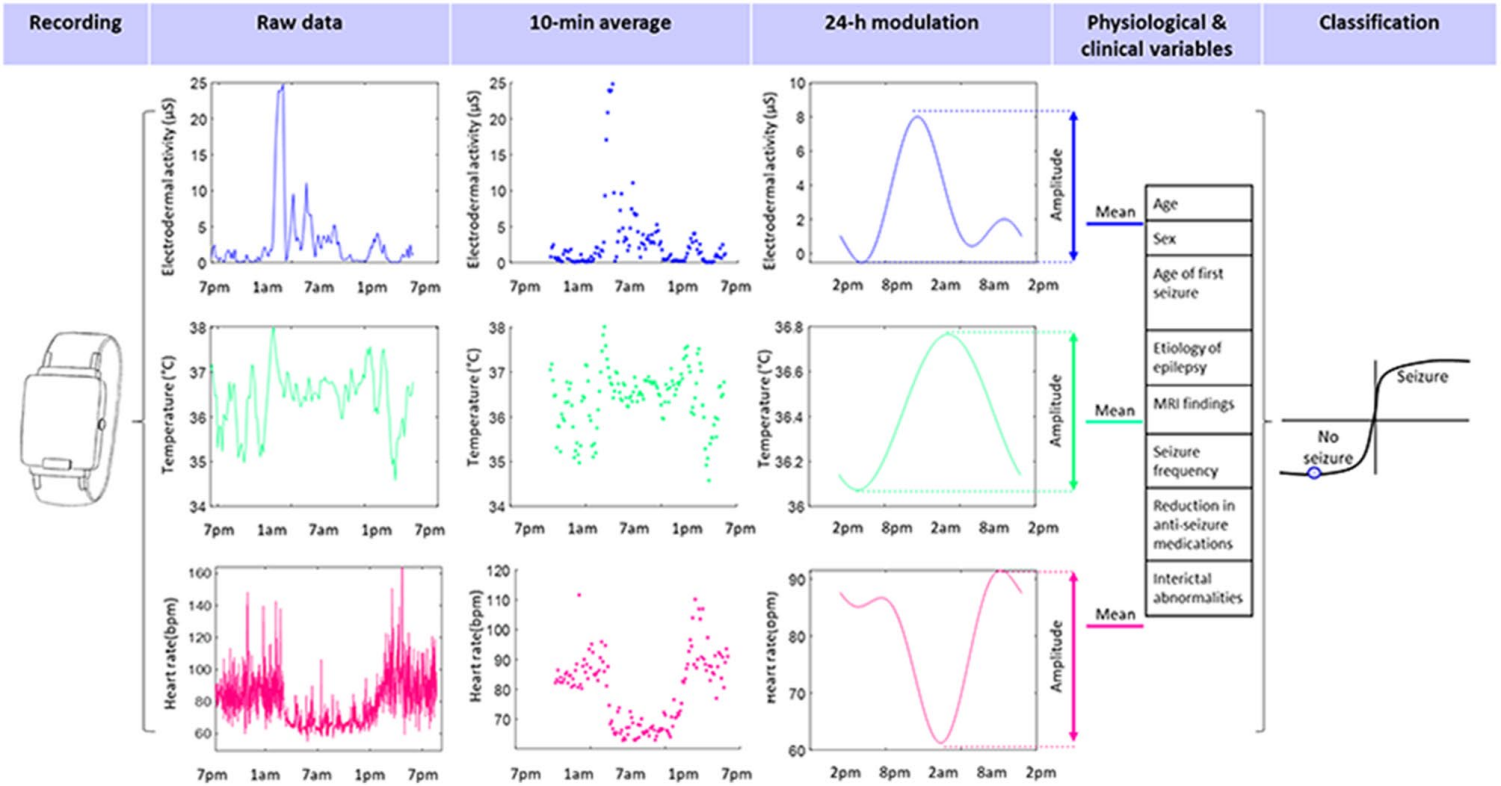
Schematic illustration of data collection and analysis steps, including (from left to right) recording with the wearable wristband, raw data processing, averaging of data over 10-min-segments, 24-h pattern modulation modeling (cycle start: 2 pm), amplitude and level calculation, adding clinical variables, and classification into a seizure or a non-seizure recording.

Data analysis was performed using MATLAB (Version R2019b, The MathWorks Inc., Natick, Massachusetts, USA). EDA, TEMP, and HR recorded values were averaged over 10-min segments for data analysis (Fig. 2). Using a nonlinear mixed-effects harmonic model^40,41^, we modeled the 24-h pattern of EDA, TEMP, and HR, by the nlinfit function implemented in MATLAB with two harmonic terms for EDA and HR and one harmonic term for TEMP. We calculated the modulation’s mean level and amplitude from the resulting curve of each patient. Two-tailed statistical tests were used and a significance level of 0.05 was predetermined. SPSS version 23 (IBM Corp., Armonk, New York, United States) was used for data analysis. We performed univariate logistic regression and tested for group differences in modulation level and amplitude of EDA, TEMP, and HR.

For classification between seizure and no seizure patients, we implemented several supervised learning algorithms from scikit-learn version 0.23.2 in Python (Python Software Foundation, Wilmington, DE, USA; version 2020.3.3)^42^. Specifically, we investigated the performance of the following five learning algorithms along with logistic regression: K-nearest neighbor, random forest, Ada Boost, Gaussian naive Bayes, and support vector machine (SVM; linear and nonlinear with Radial Basis Function (RBF) kernel). We used tenfold cross-validation and default hyperparameters of the scikit-learn toolbox. Additionally, we randomly shuffled the data labels 200 times and statistically compared the performance of the classifiers for the shuffled labels to the original labels by t-test. Code can be found here: https://doi.org/10.7910/DVN/MHU1V2. We applied Bonferroni correction to the p-values to account for multiple testing. For within-patient comparison, we performed repeated-measure ANOVA, with recording time (seizure-free recording before seizure recording and seizure recording) as the repeated factor. We excluded etiology, a nominal variable, from the classification. To verify the contribution of clinical and wearable data we ran a feature ranking and ran the same classifiers with clinical data and wearable data, leaving age and sex in both model (see Supplement 3).

## Supplementary Information

Supplementary Information 1.

Supplementary Information 2.

Supplementary Information 3.

Supplementary Information 4.

Supplementary Information 5.

## Data Availability

All statistical analyses and results are included in the manuscript. The original data are available upon reasonable request and when compatible with the IRB (please contact Tobias.Loddenkemper@childrens.harvard.edu).

## References

[1] Karoly PJ (2017). The circadian profile of epilepsy improves seizure forecasting. Brain.

[2] Karoly PJ (2020). Forecasting cycles of seizure likelihood. Epilepsia.

[3] Stirling RE, Cook MJ, Grayden DB, Karoly PJ (2021). Seizure forecasting and cyclic control of seizures. Epilepsia.

[4] Cook MJ (2013). Prediction of seizure likelihood with a long-term, implanted seizure advisory system in patients with drug-resistant epilepsy: A first-in-man study. Lancet Neurol..

[5] Sarkis RA (2015). Autonomic changes following generalized tonic clonic seizures: An analysis of adult and pediatric patients with epilepsy. Epilepsy Res..

[6] Poh M-Z (2012). Convulsive seizure detection using a wrist-worn electrodermal activity and accelerometry biosensor. Epilepsia.

[7] Chroni E, Sirrou V, Trachani E, Sakellaropoulos GC, Polychronopoulos P (2009). Interictal alterations of cardiovagal function in chronic epilepsy. Epilepsy Res..

[8] Vieluf S (2020). Autonomic nervous system changes detected with peripheral sensors in the setting of epileptic seizures. Sci. Rep..

[9] Cogan D, Birjandtalab J, Nourani M, Harvey J, Nagaraddi V (2017). Multi-biosignal analysis for epileptic seizure monitoring. Int. J. Neural Syst..

[10] Regalia G, Onorati F, Lai M, Caborni C, Picard RW (2019). Multimodal wrist-worn devices for seizure detection and advancing research: Focus on the Empatica wristbands. Epilepsy Res..

[11] Vieluf S (2021). Twenty-four-hour patterns in electrodermal activity recordings of patients with and without epileptic seizures. Epilepsia.

[12] Riganello F, Prada V, Soddu A, Di Perri C, Sannita WG (2019). Circadian rhythms and measures of CNS/autonomic interaction. Int. J. Environ. Res. Public. Health.

[13] Reppert SM, Weaver DR (2002). Coordination of circadian timing in mammals. Nature.

[14] Innominato, P. F. & Spiegel, D. Circadian rhythms, sleep, and anti-cancer treatments. *Sleep Health Soc. Aetiol. Public Health* 141 (2018).

[15] Vieluf S (2019). Peripheral multimodal monitoring of ANS changes related to epilepsy. Epilepsy Behav..

[16] Buijs RM, Escobar C, Swaab DF (2013). The circadian system and the balance of the autonomic nervous system. Handb. Clin. Neurol..

[17] Yamasaki Y (1996). Diurnal heart rate variability in healthy subjects: effects of aging and sex difference. Am. J. Physiol. Heart Circ. Physiol..

[18] Tayefeh F, Plattner O, Sessler DI, Ikeda T, Marder D (1998). Circadian changes in the sweating-to-vasoconstriction interthreshold range. Pflüg. Arch..

[19] Poh M-Z, Swenson NC, Picard RW (2010). A wearable sensor for unobtrusive, long-term assessment of electrodermal activity. IEEE Trans. Biomed. Eng..

[20] Yang Z (2018). The analysis of circadian rhythm of heart rate variability in patients with drug-resistant epilepsy. Epilepsy Res..

[21] Murugesan A (2018). Serum serotonin levels in patients with epileptic seizures. Epilepsia.

[22] Tang J (2021). Seizure detection using wearable sensors and machine learning: Setting a benchmark. Epilepsia.

[23] Zsom, A. *et al.* Ictal autonomic activity recorded via wearable-sensors plus machine learning can discriminate epileptic and psychogenic nonepileptic seizures. In *2019 41st Annual International Conference of the IEEE Engineering in Medicine and Biology Society (EMBC)* 3502–3506 (IEEE, 2019). 10.1109/EMBC.2019.8857552 PMC7821908 31946633

[24] Naganur VD (2019). The utility of an automated and ambulatory device for detecting and differentiating epileptic and psychogenic non-epileptic seizures. Epilepsia Open.

[25] Kusmakar, S. *et al.* Improved detection and classification of convulsive epileptic and psychogenic non-epileptic seizures using FLDA and Bayesian inference. In *2018 40th Annual International Conference of the IEEE Engineering in Medicine and Biology Society (EMBC)* 3402–3405 (IEEE, 2018). 10.1109/EMBC.2018.8512981 30441118

[26] Critchley HD, Harrison NA (2013). Visceral influences on brain and behavior. Neuron.

[27] Beissner F, Meissner K, Bär K-J, Napadow V (2013). The autonomic brain: an activation likelihood estimation meta-analysis for central processing of autonomic function. J. Neurosci..

[28] Macey PM, Ogren JA, Kumar R, Harper RM (2016). Functional imaging of autonomic regulation: Methods and key findings. Front. Neurosci..

[29] Behbahani S, Jafarnia Dabanloo N, Motie Nasrabadi A, Dourado A (2018). Gender-related differences in heart rate variability of epileptic patients. Am. J. Mens Health.

[30] Allen LA, Harper RM, Lhatoo S, Lemieux L, Diehl B (2019). Neuroimaging of Sudden Unexpected Death in Epilepsy (SUDEP): Insights from structural and resting-state functional MRI studies. Front. Neurol..

[31] Al-Bakri, A. F., Villamar, M. F., Haddix, C., Bensalem-Owen, M. & Sunderam, S. Noninvasive seizure prediction using autonomic measurements in patients with refractory epilepsy. In *2018 40th Annual International Conference of the IEEE Engineering in Medicine and Biology Society (EMBC)* 2422–2425 (IEEE, 2018). 10.1109/EMBC.2018.8512785 30440896

[32] Meisel C (2020). Machine learning from wristband sensor data for wearable, noninvasive seizure forecasting. Epilepsia.

[33] Yamakawa T (2020). Wearable epileptic seizure prediction system with machine-learning-based anomaly detection of heart rate variability. Sensors.

[34] Goldenholz DM (2020). Development and validation of forecasting next reported seizure using e-diaries. Ann. Neurol..

[35] Karoly PJ (2021). Cycles of self-reported seizure likelihood correspond to yield of diagnostic epilepsy monitoring. Epilepsia.

[36] Baud MO (2018). Multi-day rhythms modulate seizure risk in epilepsy. Nat. Commun..

[37] Proix T (2021). Forecasting seizure risk in adults with focal epilepsy: a development and validation study. Lancet Neurol..

[38] Karoly PJ (2021). Multiday cycles of heart rate are associated with seizure likelihood: An observational cohort study. BioMedicine.

[39] Fisher RS (2017). Instruction manual for the ILAE 2017 operational classification of seizure types. Epilepsia.

[40] Refinetti R, Cornélissen G, Halberg F (2007). Procedures for numerical analysis of circadian rhythms. Biol. Rhythm Res..

[41] Albert PS, Hunsberger S (2005). On analyzing circadian rhythms data using nonlinear mixed models with harmonic terms. Biometrics.

[42] Varoquaux G (2015). Scikit-learn: Machine learning without learning the machinery. GetMobile Mob. Comput. Commun..

