# Supplementary material for: Seizure-related differences in biosignal 24-h modulation patterns
Source: Sci Rep. 2022 Sep 5;12:15070. doi: 10.1038/s41598-022-18271-z (PMC9445076; doi:10.1038/s41598-022-18271-z)
Supplement: Supplementary file 1 — Supplementary Information 1. [file 41598_2022_18271_MOESM1_ESM.docx]

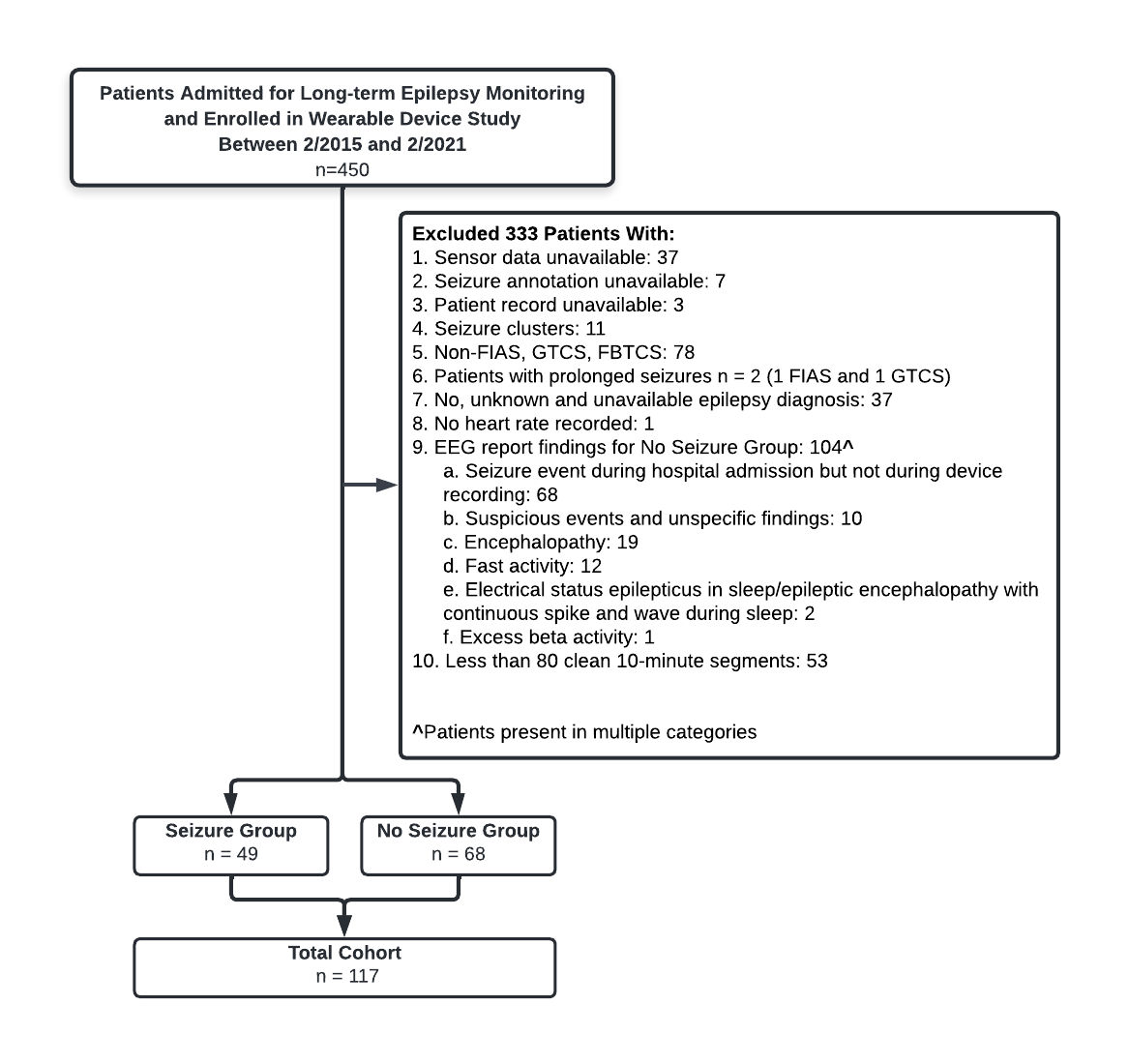


*Supplement 1: Inclusion tree depicts the data selection process from patient enrollment to study inclusion. Created in Lucidchart, www.lucidchart.com*
