# Supplementary material for: Seizure-related differences in biosignal 24-h modulation patterns
Source: Sci Rep. 2022 Sep 5;12:15070. doi: 10.1038/s41598-022-18271-z (PMC9445076; doi:10.1038/s41598-022-18271-z)
Supplement: Supplementary file 2 — Supplementary Information 2. [file 41598_2022_18271_MOESM2_ESM.docx]

*Supplement 2:* Seizure characterization for 137 seizures from 49 patients.

| Number of seizures during analyzed recordings | Mean (min-max) |
| --- | --- |
| All seizure types | 2.8 (1-11) |
| Seizure Type | Number of patients (no. of seizures) |
| GTCS | 26 (62) |
| FIAS | 8 (21) |
| Both | 15 (54) |
| Seizure Timing | Number of seizures |
| 2:00 pm to 7:59 pm | 28 |
| 8:00 pm to 1:59 am | 32 |
| 2:00 am to 7:59 am | 38 |
| 8:00 am to 1:59 pm | 39 |
| Vigilance State | Number of seizures (%) |
| Sleep | 51 (37.2) |
| Awake | 79 (57.7) |
| Not Available | 7 (5) |
| Seizure Duration* | Seconds |
| Mean (min - max) | 70.7 (2 -1378) |
| Seizure Localization and Lateralization by most frequent onset zone** | Number of seizures |
| Generalized | 31 |
| Temporal | L: 39, R: 16 |
| Frontal | L: 6, R: 9, B: 7 |
| Parietal | L: 7, R: 4, M: 2 |
| Parasagittal | R: 3 |
| Posterior | R: 2 |
| Central | L: 8, R: 5, M: 6 |
| Occipital | L: 4 |
| Hemisphere | L: 4, R: 8 |
| Unknown/Not Available | M: 2 |
| Abbreviations: GTCS, Generalized tonic-clonic seizures; FIAS, Focal impaired awareness seizures; R, right; L, left; B, bilateral; M, midline.  *Seizure duration is calculated for isolated seizures only, not clusters of seizures. The dataset contains 3 total clusters from 2 patients.  **For seizure-onset localization, we report the number of seizures in each location and the lateralization. Seizures can occur in multiple locations. Examples: Temporal (L: 2): There are two seizures with left temporal onset. Generalized (1): there is one seizure with generalized onset, and the lateralization does not apply. | |
