# Supplementary material for: Seizure-related differences in biosignal 24-h modulation patterns
Source: Sci Rep. 2022 Sep 5;12:15070. doi: 10.1038/s41598-022-18271-z (PMC9445076; doi:10.1038/s41598-022-18271-z)
Supplement: Supplementary file 3 — Supplementary Information 3. [file 41598_2022_18271_MOESM3_ESM.docx]

Supplement 3. Classifier performance for the classification between recordings of patients with and without seizures.

| Classifier*** | Accuracy | Sensitivity | Specificity | AUC ROC (CI-, CI+)* | Shuffle Accuracy (SD)** | P-value |
| --- | --- | --- | --- | --- | --- | --- |
|  | All clinical and wearable data | | | | | |
| Logistic Regression | 0.74 | 0.71 | 0.75 | 0.78 (0.76,0.80) | 0.53 (0.05) | 0.005 |
| K-Nearest Neighbors | 0.76 | 0.61 | 0.87 | 0.79 (0.76,0.82) | 0.52 (0.05) | 0.005 |
| Random Forest | 0.68 | 0.55 | 0.76 | 0.76 (0.74,0.79) | 0.53 (0.05) | 0.005 |
| Ada Boost | 0.63 | 0.55 | 0.69 | 0.62 (0.58,0.65) | 0.51 (0.05) | 0.010 |
| Naive Bayes | 0.53 | 0.96 | 0.22 | 0.79 (0.77,0.82) | 0.5 (0.06) | 0.300 |
| Linear SVM | 0.74 | 0.73 | 0.74 | 0.77 (0.75,0.79) | 0.53 (0.06) | 0.005 |
| RBF SVM | 0.74 | 0.63 | 0.81 | 0.77 (0.75,0.80) | 0.55 (0.05) | 0.005 |
| Data source | Clinical data | | | | | |
| Logistic Regression | 0.71 | 0.57 | 0.81 | 0.81 (0.78,0.83) | 0.54 (0.05) | 0.149 |
| K-Nearest Neighbors | 0.74 | 0.63 | 0.81 | 0.78 (0.76,0.80) | 0.52 (0.06) | 0.060 |
| Random Forest | 0.66 | 0.55 | 0.74 | 0.70 (0.68,0.72) | 0.53 (0.05) | 0.075 |
| Ada Boost | 0.67 | 0.53 | 0.76 | 0.72 (0.70,0.81) | 0.53 (0.05) | 0.537 |
| Naive Bayes | 0.5 | 0.98 | 0.15 | 0.81 (0.78,0.84) | 0.51 (0.06) | 0.035 |
| Linear SVM | 0.68 | 0.57 | 0.76 | 0.79 (0.77,0.81) | 0.55 (0.05) | 0.294 |
| RBF SVM | 0.69 | 0.53 | 0.81 | 0.74 (0.72,0.76) | 0.55 (0.05) | 0.224 |
| Data source | Wearable data | | | | | |
| Logistic Regression | 0.59 | 0.51 | 0.65 | 0.65 (0.76,0.80) | 0.54 (0.05) | 0.005 |
| K-Nearest Neighbors | 0.61 | 0.47 | 0.71 | 0.64 (0.76,0.82) | 0.52 (0.05) | 0.005 |
| Random Forest | 0.63 | 0.51 | 0.72 | 0.64 (0.74,0.79) | 0.53 (0.05) | 0.010 |
| Ada Boost | 0.51 | 0.49 | 0.53 | 0.46 (0.58,0.65) | 0.52 (0.05) | 0.010 |
| Naive Bayes | 0.61 | 0.82 | 0.46 | 0.66 (0.77,0.82) | 0.52 (0.05) | 0.577 |
| Linear SVM | 0.57 | 0.55 | 0.59 | 0.63 (0.75,0.79) | 0.55 (0.04) | 0.005 |
| RBF SVM | 0.59 | 0.43 | 0.71 | 0.62 (0.75,0.80) | 0.55 (0.04) | 0.005 |

**The area under the curve for receiver operating characteristics (AUC ROC);*

***200 Shuffles were performed. Bonferroni corrected level of significance was 0.007 (for significant comparisons to shuffled labels p-values are highlighted in bold).*

****Feature ranking: Optimal number of features = 15; Grid scores: EDA amplitude 0.61, EDA level 0.59, HR amplitude 0.58, HR level 0.62, TEMP amplitude 0.63, TEMP level 0.65, Sex 0.69, Age 0.70, Age at first seizure 0.69, MRI finding 0.69, ASM reduction 0.7, Normal EEG 0.72, Focal slowing 0.73, Generalized slowing 0.70, Spikes 0.73.*
