# Supplementary material for: Seizure-related differences in biosignal 24-h modulation patterns
Source: Sci Rep. 2022 Sep 5;12:15070. doi: 10.1038/s41598-022-18271-z (PMC9445076; doi:10.1038/s41598-022-18271-z)
Supplement: Supplementary file 4 — Supplementary Information 4. [file 41598_2022_18271_MOESM4_ESM.docx]

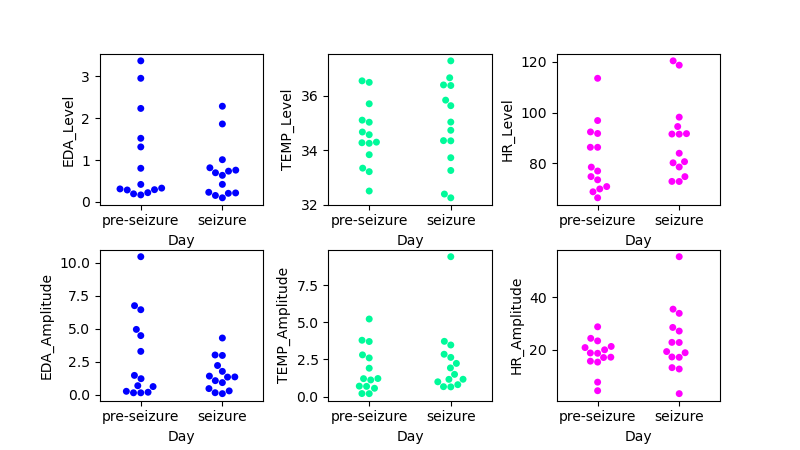


*Supplement 4:* Swarm plot illustrating electrodermal activity (EDA), peripheral body temperature (TEMP), and heart rate (HR) 24-hour modulation levels and amplitudes per patient in pre-seizure and seizure recordings.
