# Supplementary material for: Seizure-related differences in biosignal 24-h modulation patterns
Source: Sci Rep. 2022 Sep 5;12:15070. doi: 10.1038/s41598-022-18271-z (PMC9445076; doi:10.1038/s41598-022-18271-z)
Supplement: Supplementary file 5 — Supplementary Information 5. [file 41598_2022_18271_MOESM5_ESM.docx]

Supplement 5. A detailed explanation of clinical data collection variables.

| Clinical variable name | Definition | Answer options |
| --- | --- | --- |
| Age | Age at enrollment | Number in years |
| Sex | Sex at enrollment | Female  Male |
| Age at first seizure | Age at first seizure reported in neurology clinic visit and neurology admission notes. Febrile seizures are not included in this variable. This is not the age of epilepsy onset. | Number in years |
| Etiology of epilepsy | Etiology is defined as the cause of epilepsy. This variable is the etiology of epilepsy and does not refer to the etiology of seizures. This variable is not applicable if a patient is not diagnosed with an epilepsy etiology. The root cause etiology is reported if dual etiologies occur. | Structural  Unknown  Genetic  Immune  Infectious  Metabolic  Not reported |
| MRI findings | MRI clinical report findings during or before enrollment. | Normal  Abnormal  Not performed |
| Reduction of anti-seizure medications during the hospital stay | Indicates if the home medication was reduced during the hospital stay. | Yes  No |
| Seizure frequency | Calculated seizure frequency based on patient or caregiver report for the 30 days before the LTM visit | Number of seizures per 30 days |
| Normal EEG | Normal EEG is defined as no epileptiform activity and no slowing during the video-EEG monitoring. | Yes  No |
| Spikes | Indicates presence of either polyspikes, spike-wave complex, and spikes and sharp waves as determined by epileptologist review. | Yes  No |
| Focal slowing | Indicates presence of intermittent or continuous focal slowing from the EEG clinical report. | Yes  No |
| Generalized slowing | Indicates presence of intermittent or continuous generalized slowing from the EEG clinical report. | Yes  No |
